# Social effects on foraging behavior and success depend on local environmental conditions

**DOI:** 10.1002/ece3.1377

**Published:** 2015-01-04

**Authors:** Harry H Marshall, Alecia J Carter, Alexandra Ashford, J Marcus Rowcliffe, Guy Cowlishaw

**Affiliations:** 1 Institute of Zoology, Zoological Society of LondonRegent's Park, London, NW1 4RY, U.K; 2 Division of Ecology and Evolution, Department of Life Sciences, Imperial College LondonSilwood Park, Ascot, Berkshire, SL5 7PY, U.K; 3 Centre for Ecology and Conservation, University of ExeterPenryn Campus, Penryn, Cornwall, TR10 9EF, U.K; 4 The Fenner School of Environment and Society, The Australian National UniversityActon, Canberra, ACT, 0200, Australia

**Keywords:** Dominance rank, field experiment, *Papio ursinus*, Primate, social bonds, social tactics

## Abstract

In social groups, individuals' dominance rank, social bonds, and kinship with other group members have been shown to influence their foraging behavior. However, there is growing evidence that the particular effects of these social traits may also depend on local environmental conditions. We investigated this by comparing the foraging behavior of wild chacma baboons, *Papio ursinus*, under natural conditions and in a field experiment where food was spatially clumped. Data were collected from 55 animals across two troops over a 5-month period, including over 900 agonistic foraging interactions and over 600 food patch visits in each condition. In both conditions, low-ranked individuals received more agonism, but this only translated into reduced foraging performances for low-ranked individuals in the high-competition experimental conditions. Our results suggest one possible reason for this pattern may be low-ranked individuals strategically investing social effort to negotiate foraging tolerance, but the rank-offsetting effect of this investment being overwhelmed in the higher-competition experimental environment. Our results also suggest that individuals may use imbalances in their social bonds to negotiate tolerance from others under a wider range of environmental conditions, but utilize the overall strength of their social bonds in more extreme environments where feeding competition is more intense. These findings highlight that behavioral tactics such as the strategic investment of social effort may allow foragers to mitigate the costs of low rank, but that the effectiveness of these tactics is likely to be limited in certain environments.

## Introduction

In social groups, individuals vary in their dominance rank, social bonds, and kinship with other group members (Earley and Dugatkin 2010; East and Hofer 2010), all of which can have an important effect on foraging behavior and performance (Waite and Field 2007; Marshall et al. 2012a). High-ranking individuals tend to be less susceptible to interference competition and more able to initiate aggression in the defense or theft of food resources (birds: Smith et al. 2001; Liker and Barta 2002; primates: Di Bitetti and Janson 2001; Barrett et al. 2002), which in turn can facilitate access to the best patches (birds: Bautista et al. 1995; Holmgren 1999; primates: King et al. 2008). Social bonds and kinship ties with co-foragers can ameliorate these effects, particularly for subordinate animals, by reducing the aggression and/or increasing the tolerance they receive (birds: Ha et al. 2003; macropods: Carter et al. 2009; primates: King et al. 2009; Silk et al. 2010a) and allowing them to negotiate access to better patches (birds: Mathot and Giraldeau 2010; primates: Barrett et al. 1999; Fruteau et al. 2009).

While empirical evidence for the influence of social traits on foraging behavior is common, there have been far fewer empirical studies investigating how these influences may be dependent on local environmental conditions. Such dependencies are suggested by resource defense theory (Brown 1964; Grant 1993) and models of primate social evolution (reviewed by Isbell and Young 2002). In both cases, there is a general prediction that greater spatial clumping of resources should lead to a greater influence of social effects on foraging performance due to the resources being more monopolisable. The relatively small number of studies conducted to date provides support for these theories. In the case of dominance rank, for instance, Vahl et al. (2005) showed that high-ranking ruddy turnstones (*Arenaria interpres*) only experienced greater intake rates where food resources were spatially clumped. Similarly, Stillman et al. (2002) found that in common cranes (*Grus grus*) high-ranking individuals only engaged in aggressive behavior in environments where their intake fell below a certain threshold. Fewer studies have investigated the environment-dependent effects of social bonds and kinship on foraging behavior. Tanner and colleagues showed that European shore crabs (*Carcinus maenus*) only formed aggregations, and stable social networks, when food resources were clumped (Tanner et al. 2011; Tanner and Jackson 2012), whilst Nystrand (2006) found evidence that Siberian jays (*Perisoreus infaustus*) preference for foraging with their offspring (over nonrelated immigrants) was greater during high-predation risk winter months.

There is also conflicting evidence, particularly in primate systems, as to the mechanism through which social bonds influence foraging behavior, despite the general consensus that their influence can be important. Many studies highlight the role of social bond “strength”, usually indexed by the frequency or duration of bonding interactions (e.g., allogrooming), in ameliorating aggression from others or allowing access to better food patches (Frank and Silk 2009; King et al. 2009; Silk et al. 2010a). Other studies emphasize social bond “balance”, usually indexed by the relative contribution of each social partner to bonding interactions. Here, social bond imbalances occur when one partner contributes more to affiliative interactions in exchange for reduced aggression and increased foraging tolerance from the other (Barrett et al. 1999; Fruteau et al. 2009; Tiddi et al. 2011; biological market theory: Noë and Hammerstein 1995). For example, Fruteau et al. (2009) were able to increase the proportion of grooming interactions low-ranked vervet monkeys (*Chlorocebus aethiops*) received from other group members by giving them the ability to provide others with access to resources by “opening” a box of food (which the researchers remotely unlocked only when the low-ranked individual approached it).

The environment-dependent influences of rank, social bonds, and kinship on social foraging behavior are likely to play a fundamental role in determining how fitness varies between individual group members in different environments. For example, there is growing evidence that the effect of rank on reproductive success may be particularly important when foraging competition is high, such as during periods of low food availability (Altmann and Alberts 2003; Nichols et al. 2012; Clutton-Brock and Huchard 2013). Understanding these influences, and the mechanisms through which they act, is required to assess the individual costs and benefits of sociality across environments, and thus how sociality evolves. Further, such knowledge will also help us to predict how social animals are likely to be influenced by future environmental change.

We conducted this study on wild chacma baboons (*Papio ursinus)*. Baboons (*Papio* spp.) make ideal subjects for this study as they live in stable groups with well-defined dominance hierarchies and individuals vary considerably in both the strength and balance of their social bonds as well as their genetic relatedness to other group members (Cheney and Seyfarth 2008). They also live in a wide range of habitats across sub-Saharan Africa (Stone et al. 2013), including in urban environments (e.g., Hoffman and O'Riain 2012), and have been shown to flexibly adjust their social and foraging behavior in response to differing environments (Barrett et al. 2002; Alberts et al. 2005; King et al. 2009). We recorded baboon foraging behaviors in two different environments: a natural environment, where food resources occurred in many discrete patches that varied in both quality and distribution; and in a field-experimental environment, where food resources were available in a few concentrated patches. An experimental approach was adopted to broaden the range of environmental conditions explored, and in response to the growing appreciation that environmental extremes can have an important impact on animal populations (e.g., McFarland and Majolo 2013; reviewed in Ameca y Juárez et al. 2012). Our experiment represents conditions where food resources are highly spatially clumped, stimulating high levels of competition, which are often typical of periods of food scarcity.

Our primary purpose in this study was to test whether the effects of rank, social bonds, and kinship on foraging behavior are dependent on the local environment. In general, we predicted that individuals' social traits, particularly their dominance rank, would have a greater effect on their foraging behavior in the experiment, where food resources were more spatially clumped, than under natural conditions. At the same time, we also used this opportunity to assess the alternative hypotheses about the roles of social bond strength versus balance in foraging behavior (e.g., Fruteau et al. 2009; Silk et al. 2010a). Comparing between the natural and experimental environments, we explored how four social traits (rank, social bond strength, social bond balance, and kinship) influenced two measures of individuals' foraging behavior: (1) feeding-related agonism and (2) foraging performance. In the first case, we explored how individuals differed in the rate of agonism they experienced (both initiated and received) and the proportion of this agonism that they received. In the second case, we explored how individuals' social traits influenced three measures of foraging performance: (i) initial intake rate, (ii) time spent in a food patch, and (iii) the correlation between initial intake rate and patch residency time, an estimate of an individual's ability to efficiently exploit the environment (see Methods for details).

## Methods

### Study site

Fieldwork occurred at Tsaobis Leopard Park, Namibia (22°23′S, 15°45′E), from May to September 2010. The environment at Tsaobis predominantly consists of two habitats: open desert and riparian woodland. The open desert, hereafter “desert”, consists of alluvial plains and steep-sided hills mainly containing small herbs and dwarf shrubs such as *Monechma cleomoides*, *Sesamum capense,* and *Commiphora virgata*. The riparian woodland, hereafter “woodland”, is associated with the ephemeral Swakop River that bisects the site and mainly contains large trees and bushes such as *Faidherbia albida*, *Prosopis glandulosa,* and *Salvadora persica* (see Cowlishaw and Davies 1997 for more details). Baboons are omnivorous but at Tsaobis their diet predominantly consists of berries, pods, flowers, and young leaves (Cowlishaw 1997). Tsaobis baboons experience relatively low-predation risk as their main predator, the leopard (*Panthera pardus*) occurs at low densities (Cowlishaw 1994). At Tsaobis, two troops of chacma baboons (total troop sizes = 41 and 33 in May 2010), hereafter the “large” and “small” troop, have been habituated to the presence of human observers at close proximity. We collected data from all adults and those juveniles over 2 years old (*n* = 32 and 23), all of whom were individually recognizable (see Huchard et al. 2010 for details). Younger animals were not individually recognizable and so were not included in this study.

### Data collection

#### Natural foraging behavior

We recorded baboon behavior under natural conditions on handheld Motorola MC35 and Hewlett-Packard iPAQ Personal Digital Assistants using a customized spreadsheet in SpreadCE version 2.03 (Bye Design Ltd. 1999) and Cybertracker v3.237 (http://cybertracker.org), respectively. We selected focal animals in a stratified manner to ensure even sampling from four three-hour time blocks (6–9 a.m., 9 a.m.–12 p.m., 12–3 p.m. and 3–6 p.m.) across the field season. No animal was sampled more than once per day. We conducted 30-min focal follows (Altmann 1974) and discarded any lasting <20 min. At all times, we recorded the focal animal's activity (mainly foraging, resting, traveling, or grooming) and the occurrence, partner identity, and direction of any grooming or agonistic interactions. We also recorded the duration of grooming bouts and the context of agonistic interactions (e.g., access to food, water, or a preferred grooming partner). The following five behaviors, with definitions, were classed as agonistic behaviors. *Supplant*: animal A approaches animal B, causing B to move off while A takes B's position at a resource. *Displace*: A moves near to B, causing B to monitor A and/or show A subordinance (e.g., baring teeth) and to immediately move position or change direction of travel. *Threat*: A head-bobs and/or paws the ground whilst looking at B. *Chase*: A runs after B (but does not make physical contact with B). *Attack*: A makes aggressive physical contact with B, often biting or holding B to the ground.

During foraging, we recorded the focal animal's patch residency time as the time from entering to exiting a discrete food patch. Entry was defined as the focal animal moving into a patch and looking for food in it (to rule out the possibility that they were simply passing by or through the patch), and exit defined as the focal animal moving out of the patch. Patches were defined as individual herbs, shrubs or trees, or groups of conspecific neighboring plants within 1 m of each other and so within reach of the forager without moving. At each patch entry, we recorded the habitat (woodland or desert), the patch's type, size, and food-item handling time, the number of other baboons already occupying the patch and, where possible, the focal individual's initial intake rate (bites in the first ten seconds). Other data collection requirements meant it was only possible to collect one intake rate per patch visit, recorded on patch entry. We recorded patch type by species for large trees and bushes in the woodland, and otherwise as nonspecified “herb/shrub” for smaller plants in both habitats. Patch size was scored on a scale of 1–6 for large trees and bushes in the woodland and 1–4 for herb/shrubs, and subsequently converted into an estimate of surface area (m^2^) (for details of these conversions, see Marshall et al. 2012b). Food-item handling time was classed as long (bark, pods, and roots) or short (young leaves, berries, and flowers). We excluded from our analyses those natural foraging data that were collected on the same days as a feeding experiment was being run on that focal animal's troop (see below). Overall, we recorded 444 h of feeding behavior (8 ± 3 h, mean ± SD, per individual). For our analyses, this sample contained 991 agonistic interactions over food resources (18 ± 9 per individual), 2106 intake rates (39 ± 30), and 1768 patch residency times (33 ± 24) (the difference in intake rate and patch residency time sample sizes being due to the availability of the explanatory variables used in the analysis of each, see Table1).

**Table 1:** Summary of all the variables we included in our analyses with definitions where necessary. The variables are divided into those variables which were of interest to this study and those that were included in our analyses as controls. Each of the four models listed were fitted separately to natural and experimental data

Variable	Definition	Included in models of			
		Aggression rate^1^	Proportion of aggression received^1^	Intake rate^2^,^3^	Patch residency time^2^,^3^
Variables of interest					
Rank	Position within troop dominance hierarchy. See Methods text for details of calculation	All models			
Social bond strength	Mean proportion of focal follow time an individual was observed in grooming interactions	All models			
Social bond balance	Mean proportion of time spent grooming as the groomer	All models			
Relatedness	Mean dyadic relatedness to all other troop members	All models			
Rank × Social bond strength		All models			
Rank × Social bond balance		All models			
Rank × Relatedness		All models			
Social bond strength × Relatedness		All models			
Social bond balance × Relatedness		All models			
Social bond strength × Social bond balance		All models			
Initial intake rate	Number of bites during first 10 s of patch visit	No	No	No	Yes
Initial intake rate × Rank		No	No	No	Yes
Initial intake rate × Social bond balance		No	No	No	Yes
Initial intake rate × Social bond strength		No	No	No	Experiment only
Control variables					
Individual					
Age	Age at the start of the study period, estimated by tooth eruption and wear patterns. See Methods text.	All models			
Sex		All models			
Satiation	Cumulative time the individual had spent on other patches prior to visiting the current patch (s)	No	No	Experiment only	Experiment only
		No	No		
Patch					
Quality	Natural: patch surface area (m^2^); Experiment: Initial density of food (g/m^2^)	No	No	Yes	Yes
Depletion	Cumulative baboon-seconds the patch had been used for prior to the current patch visit	No	No	Experiment only	Experiment only
Species		No	No	Natural only	Natural only
Food handling time	Binary high/low (0/1). See Methods text	No	No	Natural only	Natural only
Number of occupants		No	No	Yes	Yes
Number of occupants^2^		No	No	Yes	Yes
Residency time in previous patch^4^		No	No	No	Yes
Habitat					
Mean patch quality	Natural: Mean number of food items per patch; Experiment: Mean density of food across total feeding area (g/m^2^)	All models			
Patch density	Natural: Number of patches per km^2^; Experiment: Inter-patch distance (m)	All models			

Random intercepts included in the models:

1 Focal animal ID, nested in troop ID;

2 Natural intake and patch residency time models: focal follow number, nested in focal animal ID nested in troop ID;

3 Experiment intake and patch residency time models: Focal animal ID, experimental patch ID and experiment day all cross-classified, and nested in troop ID.

4 Residency time in the previous patch was included in our models on patch residency time as a previous study at this site showed this can be an important predictor (Marshall et al. 2013).

For each habitat in each month, we estimated both the mean number of food items per patch, and the patch density for each habitat, using monthly phenological surveys. In these surveys, a pair of observers visited a random subset of potential patches in each habitat, estimated by eye the number of food items in the patch, and then recorded the mean of their estimates. In the woodland, these were a representative sample of 110 patches from an earlier survey of 5693 woodland patches (G. Cowlishaw, unpubl. data). In the desert, these were 73 food patches that fell within eight 50 × 1 m transects randomly placed around four localities within the study troops' home ranges. Patch density estimates were calculated as the mean number of these potential patches which contained food per km^2^.

#### Large-scale foraging experiment

**Figure 1:**
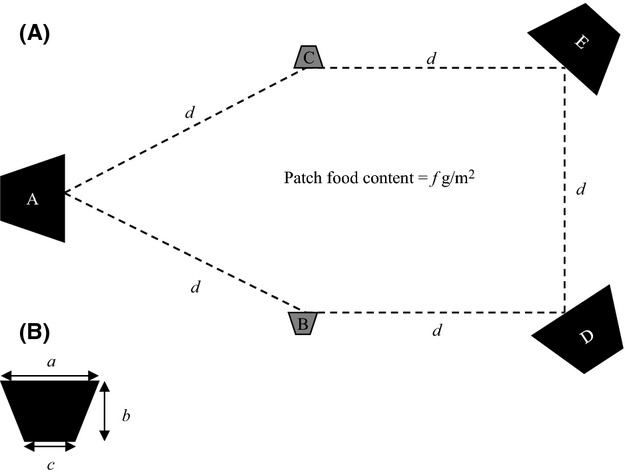
Schematic of the foraging experiment's patch (A) layout and (B) dimensions. For each troop, patch food content, *f*, was varied between 11.4 ± 0.3 g/m^2^ (low, first 14-day period) and 17.1 ± 0.3 g/m^2^ (high, second 14-day treatment) of loose dried maize kernels. Interpatch distance (*d*) was varied with each 14-day period. In the first period, it was set at 25 m (short) for the starting 7 days and 50 m (long) for the remaining 7 days, and vice versa for the second period. Patch size was constant within troops. Large patches (A, D and E) were set at 80 m^2^ (a = 10 m, b = 10 m, c = 6 m) for the small troop and 96 m^2^ (10, 12, 6) for the large troop. Small patches (B and C) were set at 20 m^2^ (5, 5, 3) for the small troop and 27 m^2^ (6, 6, 3) for the large troop.

We conducted foraging experiments in an open, flat, and sandy area in each troop's home range. They involved a configuration of five artificial food patches of loose maize kernels arranged as shown in Figure1. We selected maize kernels as the experimental food as they are uniform and small in size, and previous experience at the field site has shown that baboons will usually completely consume kernels individually (one kernel per bite) at the feeding arena rather than carrying them elsewhere (King et al. 2008; Marshall et al. 2013). We based the total feeding area (all patches combined) on previous experience at the study site of the per-animal feeding area required to allow all troop members to access the foraging experiment (King et al. 2008). We determined the largest individual patch sizes by the maximum area that one camera could record (and so the size one patch could be). The five patches were a combination of sizes, two measuring 20 m^2^ (patches B and C in Fig.1), and three measuring 80 m^2^ (patches A, D, and E) for the small troop, producing a total per-animal feeding area of 8.5 m^2^ (280 m^2^ divided by 33 animals). We kept the total per-animal feeding area approximately constant by increasing these patch sizes to 27 and 96 m^2^ for the large troop, producing a total per-animal feeding area of 8.3 m^2^ (342 m^2^ divided by 41 animals). We ran the experiment in two 14-day periods for each troop (small troop: 6/6/10 to 19/6/10 and 12/8/10 to 25/8/10; large troop; 30/6/10 to 13/7/10 and 31/8/10 to 13/9/10). In the first period, patch food content (*f* in Fig.1) was “low” (11.4 ± 0.3 g/m^2^, mean ± SD) while interpatch distance (*d*) was “short” (25 m) for the first 7 days and “long” (50 m) for the second 7 days. In the second 14-day period, patch food content was increased by 50% to “high” (17.1 ± 0.4 g/m^2^) while interpatch distance was “long” for the first 7 days and “short” for the second 7 days. The experiments were therefore run over 28 days in total, involving four different food content/interpatch distance combinations, for each troop.

Experimental food patches were marked out with large stones, painted white, and were evenly scattered with maize kernels before dawn each morning. Panasonic SDR-S15 video cameras (one per patch, started simultaneously when the first baboon was sighted and stopped when the last baboon left the experimental patches) were used to record all patch activity while trained observers (one per patch) narrated the individual foragers' identities. Patch entry and exit data were subsequently transcribed from the videos to create a dataset in which each row represented one patch visit and included the following: the patch residency time (s), the initial food density of the patch at the start of the experiment (g/m^2^), and the number and identity of all other individuals in the patch. Direct measures of patch depletion and the baboons' levels of satiation were not available. Therefore, patch depletion was estimated as the cumulative number of seconds any baboon had previously occupied the patch, and level of satiation was estimated as the cumulative number of seconds the focal baboon had foraged in any patch that day (the baboons visited the experimental patches at the start of the day before natural foraging). A random selection of these patch visits was then selected and, for comparability with the data recorded under natural conditions, the initial intake rate (bites in the first ten seconds) recorded. We excluded data from day 11 of the large troop's experiment due an error with some of the video cameras. Each experiment lasted a mean of 70 min per day (SD = 21, *n* = 56) and produced a mean of 9 ± 7 h (mean ± SD) of feeding behavior per individual. For our analyses, this contained 26,431 agonistic interactions (645 ± 471 per individual), 737 intake rates (18 ± 8), and 627 patch residency times (16 ± 8) (again, the difference in intake rate and patch residency time sample sizes being due to the availability of the explanatory variables used in the analysis of each, see Table1).

#### Individual forager characteristics

We calculated each focal animal's dominance rank, social bond strength, social bond balance, and genetic relatedness to other animals in the troop. We calculated dominance hierarchies from dominance interactions recorded in the focal follows and *ad libitum* (in both cases, outside of the experimental periods; *n*_large_ = 2391, *n*_small_ = 1931) using Matman 1.1.4 (Noldus Information Technology 2003). Hierarchies in both troops were strongly linear (Landau's corrected linearity index: *h*'_large_ = 0.71, *h*'_small_ = 0.82, *P *<* *0.001 in both). As we were interested in the effect of rank within a group, rather than differences in this effect between groups, we standardized these hierarchies to vary between 0 (lowest ranked) and 1 (highest ranked) to control for the difference in troop sizes. Social bond strength was measured as the mean social bond strength across all possible dyads in the group, where each was calculated as the proportion of an individual's total focal time that it was observed grooming in that dyad. Social bond balance was calculated as the mean of the proportion of this grooming time with each other group member that the individual was the groomer minus 0.5 (to make balanced relationships equal to 0) and so described an individual's mean contribution to its social relationships. A high balance score (above 0) represented individuals who contributed a disproportionate amount of grooming time which they might then be able to exchange for reduced aggression or increased foraging tolerance (Noë and Hammerstein 1995; Fruteau et al. 2009). Finally, relatedness was measured as individuals' mean relatedness coefficient (*r*) to all other individuals in the troop, which was estimated on the basis of 16 microsatellite loci using Wang's triadic estimator (Wang 2007), see Huchard et al. (2010) for further details. Individuals' ages were estimated from tooth eruption and wear patterns (Kahumbu and Eley 1991; Huchard et al. 2009), except in two cases (one per troop) where the actual birth dates were known.

### Analysis

Prior to our full analysis, we verified that our experimental design had intensified feeding competition. To do this, we compared the rates of food-related agonism individuals experienced in natural and experimental conditions using a Wilcoxon signed-rank test.

We conducted our full analysis in two stages in which we used generalized linear mixed models (GLMMs) to compare how social traits predicted measures of individuals' (1) feeding-related agonism and (2) foraging performance, under the natural and experimental conditions. Table1 details the explanatory variables of interest that we included in each model (e.g., rank, social bond strength, social bond balance, and kinship), those that we included as control variables (e.g., individual age, number of patch occupants, patch species) and those that we included as random effects (e.g., individual and group ID). Correlations between the four social traits (rank, social bond strength, social bond balance, and kinship) were below the levels that Freckleton (2011) showed were likely to lead to biased parameter estimates and inflated variance estimates in linear model fitting (Spearman's *rho* range = −0.44 to 0.27, *n* = 55 individuals; within troop, large troop: −0.44 to 0.41, *n* = 32, small troop: −0.48 to 0.27, *n* = 23). For each response variable, we fitted separate models to data recorded under natural and experimental conditions.

In the first stage of our analysis, we explored how social traits predicted two measures of feeding-related agonism: (i) the rate of feeding-related agonistic interactions in which an individual was involved (calculated per month and habitat in natural conditions and per patch configuration in experimental conditions) and (ii) the proportion of these interactions that they received. We fitted models predicting agonism rates using a compound Poisson error structure with a log link as these data were overdispersed and also continuous. We fitted models predicting the proportion of agonism initiated using a binomial error structure and a logit link function.

In the second stage, we explored how social traits predicted individuals' foraging performance as measured by: (i) initial intake rate upon entering a patch and (ii) patch residency time. We fitted models predicting initial intake rate and patch residency time using an observation-level random effect and a Poisson-lognormal error structure with a log-link function (Elston et al. 2001) as both were overdispersed.

We also explored how social traits predicted foragers' ability to efficiently exploit the environment. To do this, we included initial intake rate plus the interactions between intake rate and the important variables identified by our agonism models as fixed effects in the models of patch residency time (see Table1). The inclusion of initial intake rate was based on the assumption (derived from foraging theory) that the optimal strategy for exploiting an environment involves leaving patches once their food content falls below a fixed threshold (Charnov 1976; Stephens and Krebs 1986). This intake rate maximization strategy should lead to a positive correlation between initial intake rate and residency time (after controlling for physical and social factors influencing differences in patch depletion rates). The inclusion of the interaction between initial intake rate and the important variables identified by our agonism models allows us to consider the potential complication that using intake rate maximization as a measure of optimality ignores other important fitness-linked factors, such as foraging aggression. These factors are likely to vary between individuals in social groups, influencing the maximum intake rate they can achieve and so their ability to exploit their environment (Nonacs 2001).

In both stages, all continuous explanatory variables were standardized to have a mean of zero and standard deviation of one to aid model fitting and allow the comparison of effect sizes (Schielzeth 2010). Model selection for each analysis was carried out using an information-theoretic (IT) approach which is increasingly recommended over more conventional methods such as stepwise regression or use of the full model in complex behavioral analyses such as these (Whittingham et al. 2006; Burnham et al. 2011; Richards et al. 2011; but see Hegyi and Garamszegi 2011; for discussion of issues in IT model selection). For each of our models, the candidate model set consisted of all possible combinations of the explanatory variables of interest detailed in Table1. The control variables listed in Table1 were retained in all candidate models. Following Burnham and Anderson (2002), candidate models in the agonism analyses, the first stage of our analyses, were evaluated using AIC_c_ because *n*/*k *<* *40 in all cases (where *n* is the number of observations, and *k* is the number of parameters in the maximal model) whereas candidate models in the foraging analyses, the second stage of our analyses, were evaluated using AIC because *n*/*k *>* *40 in all cases. The maximum Akaike's model weight was relatively low in all analyses (maximum weight = 0.24) meaning one model from each analysis' candidate set could not be selected with certainty. We therefore used all-subset model averaging, following Symonds and Moussalli (2011), to calculate a final model for each analysis. We interpreted the influence of each variable on the basis of both their parameter estimate (and associated confidence interval) and Akaike's variable importance.

All analyses were performed in R version 3.02 (R Core Team 2013) using the cplm package version 0.7–2 to fit compound Poisson models (Zhang 2014), the lme4 package version 1.1–7 to fit all other GLMMs (Bates et al. 2014) and the MuMIn package version 1.10.5 for model averaging (Barton 2014).

## Results

The rate of agonism experienced by baboons in natural foraging conditions (median = 0.030 interactions/minute, interquartile range = 0.021–0.041, *n* = 54 individuals) was lower than in experimental conditions (1.37 interactions/minute, 0.83–1.76, *n* = 41 individuals; paired Wilcoxon signed-rank test *W *=* *861, *P* < 0.0001, *n* = 41). The rate of agonism under natural conditions were comparable to rates of baboon foraging competition under natural conditions reported elsewhere (e.g., Shopland 1987: 0.145 interactions per minute; Barton 1993: 0.002–0.023; Barrett et al. 2002: 0.012–0.037). The higher rate of agonism under experimental conditions confirmed that we had successfully manipulated the level of foraging competition and bore comparison with rates of competition recorded at artificially clumped food resources in baboons (Barton 1993: 0.048–0.89 interactions per minute).

### Feeding-related agonism

**Figure 2:**
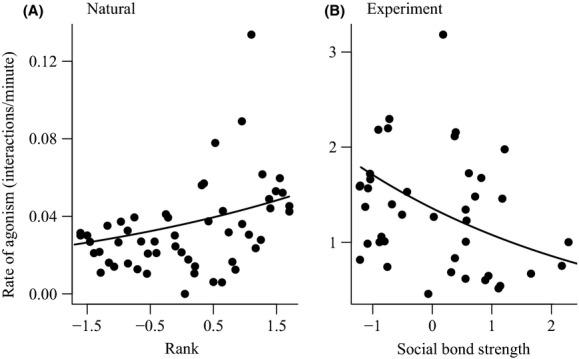
The effect of individuals' rank in natural conditions (A) and social bond strength in experimental conditions (B) on the rate of agonism they experienced. In all plots, the points are median values for each individual (calculated from the repeated measures in the data for illustrative purposes) and the lines are the predicted relationships from each model (see Table2). Values on the *x*-axes were standardized to have a mean of zero and standard deviation of one before model fitting.

The first stage of our analysis explored how social traits predicted measures of feeding-related agonism in each environment (Tables2 and 3). The rate of agonism individuals experienced increased with dominance rank under natural conditions (Fig.2A) but rank only had a weak (and negative) effect in the experiment (Table2). Instead, the rate of agonism experienced in the experiment was most strongly predicted by an individual's social bond strength (Table2): individuals who spent more time in grooming bouts with social partners experienced lower rates of agonism (Fig.2B).

**Table 2:** All-subset averaged models describing the predictors of the rate of agonism individuals experienced in natural and experimental foraging conditions. Importance is measured by Akaike's variable weight

Fixed effects	Natural				Experimental			
	Coefficient	Upper C.I.	Lower C.I.	Importance	Coefficient	Upper C.I.	Lower C.I.	Importance
(Intercept)	−3.34	−3.68	−3.01		0.70	0.27	1.13	
Rank	**0.20**	**0.06**	**0.33**	**0.97**	−0.12	−0.26	0.02	0.65
Social bond strength	−0.05	−0.23	0.14	0.44	−**0.23**	−**0.44**	−**0.02**	**0.81**
Social bond balance	−0.04	−0.18	0.10	0.51	0.04	−0.19	0.27	0.38
Relatedness	−0.06	−0.17	0.05	0.57	0.05	−0.07	0.18	0.43
Rank × Social bond strength	−0.02	−0.16	0.12	0.11	0.00	−0.14	0.14	0.12
Rank × Social bond balance	0.09	−0.03	0.20	0.24	0.07	−0.08	0.22	0.07
Rank × Relatedness	−0.07	−0.18	0.05	0.22	0.02	−0.10	0.14	0.06
Social Bond Strength × Relatedness	−0.03	−0.15	0.09	0.08	−0.05	−0.17	0.07	0.10
Social Bond Balance × Relatedness	0.01	−0.11	0.14	0.08	0.03	−0.10	0.15	0.04
Social bond strength × Social bond balance	−0.01	−0.16	0.14	0.06	−0.04	−0.20	0.13	0.08
Age	−0.01	−0.04	0.03		−0.05	−0.09	0.00	
Sex (male)^1^	0.24	−0.14	0.62		−0.22	−0.74	0.31	
Mean patch quality	−0.01	−0.12	0.11		−0.21	−0.30	−0.12	
Patch density	−0.15	−0.27	−0.04		−0.06	−0.14	0.02	

1 Reference category = female; Bold variables are those with an importance ≥0.80 and confidence intervals that do not cross zero.

**Table 3:** All-subset averaged models describing the predictors of proportion of agonism during foraging that individuals received during foraging in natural and experimental foraging conditions. Importance is measured by Akaike's variable weight

Fixed effects	Natural				Experimental			
	Coefficient	Upper C.I.	Lower C.I.	Importance	Coefficient	Upper C.I.	Lower C.I.	Importance
(Intercept)	0.48	−0.31	1.27		0.01	−0.91	0.93	
Rank	−**1.32**	−**1.64**	−**0.99**	**1.00**	−**1.37**	−**1.66**	−**1.09**	**1.00**
Social bond strength	−0.07	−0.49	0.34	0.45	0.22	−0.18	0.62	0.97
Social bond balance	0.05	−0.30	0.39	0.87	−0.12	−0.58	0.33	1.00
Relatedness	−0.07	−0.31	0.17	0.51	−0.21	−0.44	0.03	0.73
Rank × Social bond strength	0.02	−0.35	0.39	0.12	−**0.53**	−**0.84**	−**0.22**	**0.95**
Rank × Social bond balance	**0.41**	**0.11**	**0.71**	**0.80**	**0.81**	**0.47**	**1.16**	**1.00**
Rank × Relatedness	0.16	−0.13	0.45	0.19	−0.13	−0.40	0.13	0.25
Social Bond Strength × Relatedness	0.11	−0.16	0.37	0.08	0.10	−0.14	0.35	0.21
Social Bond Balance × Relatedness	0.07	−0.22	0.37	0.13	−0.08	−0.37	0.21	0.19
Social bond strength × Social bond balance	0.10	−0.23	0.42	0.11	−0.04	−0.48	0.39	0.24
Age	−0.02	−0.10	0.06		0.03	−0.06	0.12	
Sex (male)^1^	0.19	−0.73	1.11		−0.09	−1.06	0.89	
Mean patch quality	−0.25	−0.44	−0.07		−0.20	−0.23	−0.16	
Patch density	−0.24	−0.43	−0.04		−0.04	−0.07	−0.01	

1 Reference category = female; Bold variables are those with an importance ≥0.80 and confidence intervals that do not cross zero.

**Figure 3:**
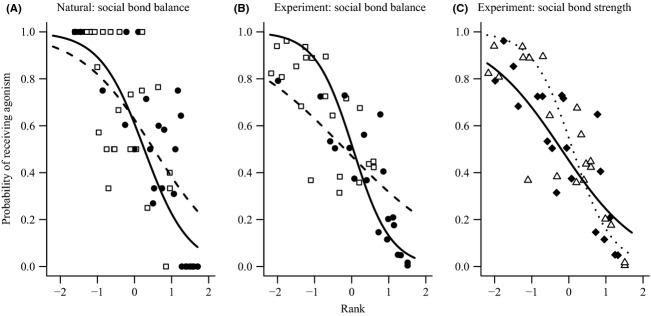
The effect of individuals' rank and social bonds on the proportion agonistic interactions they were recipient of in natural (A) and experimental conditions (B and C). In all plots, the points are median values for each individual (calculated from the repeated measures in the data for illustrative purposes) and the lines are the predicted relationships from each model (see Table3). In plots A and B, individuals are divided into those with higher (open squares and dashed line) and lower (closed circles and solid line) social bond balance than the median. In plot C, individuals are divided by social bond strength in to those with higher (open triangle and dotted line) and lower (closed diamonds and solid line) than the median. Values on the *x*-axes were standardized to have a mean of zero and standard deviation of one before model fitting.

The proportion of agonistic interactions that individuals received ranged from 0 to 1 (median = 0.59, *n* = 54) in natural conditions and from 0.04 to 0.99 (median = 0.53, *n* = 41) in experimental conditions. Higher-ranked animals were less likely to be the recipients of agonistic interactions in both conditions, but in both cases an individual's social bonds mediated this relationship (Table3). In natural conditions, high social bond balance reduced the probability of low-ranked animals receiving agonism and increased it for high-ranked animals; however, the size and importance of this effect was moderate (Table3; Fig.3A). The effect of social bond balance was similar but stronger and more important in the experiment (Table3; Fig.3B). Social bond strength also mediated the effect of rank on the probability of receiving agonism, but only in the experiment. In this case, high social bond strength increased the probability of low-ranked animals receiving agonism but reduced it in high-ranked animals (Fig.3C).

### Foraging performance

**Figure 4:**
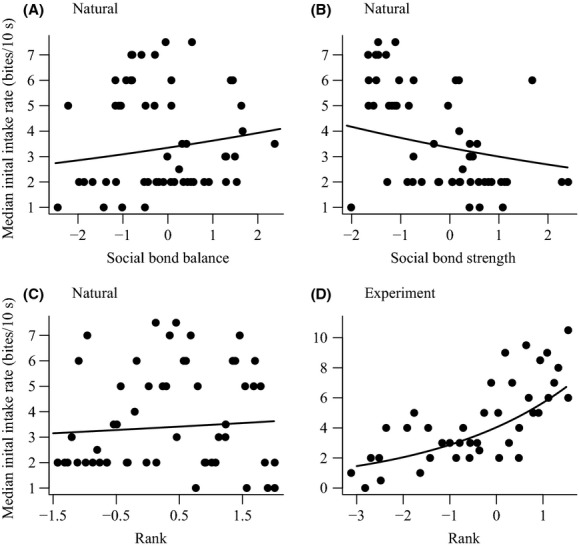
The important social traits predicting individuals' initial intake rate (bites in first seconds of patch visit) in natural (A, B, and C) and experimental conditions (D). In all plots, the points are median values for each individual and lines are the relationship estimated by each model (see Table4). Values on the *x*-axes were standardized to have a mean of zero and standard deviation of one before model fitting.

The second stage of our analysis explored how social traits predicted individual foraging performance in each environment (Tables4 and 5). The baboons experienced median intake rates of 3 bites/10 s (interquartile range = 2–6, *n* = 2106) in natural conditions and 4 bites/10 s (1–9, *n* = 737) in experimental conditions. Social bond balance and strength were both important predictors of intake rates in natural conditions (Table4). Individuals with high social bond balance experienced higher intake rates (Fig.4A), but individuals with high social bond strength experienced lower intake rates (Fig.4B). Rank also weakly predicted intake rates in natural conditions (Table4), with higher-ranked individuals experiencing slightly higher intake rates (Fig.4C). However, although important, the effect sizes of rank and social bonds strength and social bond balance were small and the confidence intervals associated with the effects of rank and social bond balance crossed zero (Table4). In contrast, in the experiment rank was an important and strong predictor of intake rates (Table4), with higher-ranked individuals experiencing considerably higher intake rates here (Fig.4D).

**Table 4:** All-subset averaged models describing the predictors of individuals' intake rate during foraging in natural and experimental foraging conditions. Importance is measured by Akaike's variable weight

Fixed effects	Natural				Experimental			
	Coefficient	Upper C.I.	Lower C.I.	Importance	Coefficient	Upper C.I.	Lower C.I.	Importance
(Intercept)	0.44	0.09	0.80		1.32	0.83	1.82	
Rank	0.04	−0.06	0.14	0.88	**0.32**	**0.18**	**0.46**	**1.00**
Social bond strength	−**0.11**	−**0.22**	−**0.01**	**0.92**	0.06	−0.15	0.27	0.66
Social bond balance	0.08	−0.01	0.17	0.95	0.05	−0.18	0.28	0.76
Relatedness	−0.04	−0.11	0.03	0.74	−0.03	−0.18	0.12	0.76
Rank × Social bond strength	0.00	−0.10	0.10	0.24	−0.10	−0.23	0.03	0.35
Rank × Social bond balance	0.09	0.02	0.16	0.72	−0.10	−0.23	0.03	0.40
Rank × Relatedness	−0.07	−0.15	0.01	0.39	−0.06	−0.19	0.06	0.33
Social Bond Strength × Relatedness	0.04	−0.05	0.13	0.25	0.07	−0.05	0.18	0.20
Social Bond Balance × Relatedness	−0.07	−0.15	0.00	0.42	0.11	0.00	0.23	0.41
Social bond strength × Social bond balance	−0.04	−0.12	0.03	0.37	0.06	−0.12	0.24	0.17
Age	−0.02	−0.04	0.00		0.01	−0.04	0.06	
Sex (male)^1^	0.23	−0.09	0.55		0.09	−0.37	0.55	
Satiation	–	–	–		−0.31	−0.41	−0.22	
Patch quality	0.00	−0.07	0.07		−0.26	−0.53	0.01	
Patch depletion	–	–	–		−0.32	−0.42	−0.22	
Patch species^2^	–	–	–		–	–	–	
*Faidherbia albida*	0.21	−0.22	0.63		–	–	–	
Herb/shrub	0.65	0.08	1.21		–	–	–	
*Prosopis glandulosa*	0.54	0.14	0.95		–	–	–	
*Salvadora persica*	0.69	0.12	1.27		–	–	–	
*Tapinanthus oleifolius*	0.36	−0.22	0.94		–	–	–	
*Acacia tortilis*	−0.44	−1.48	0.60		–	–	–	
Handling time (low)^3^	0.21	−0.27	0.69		–	–	–	
No. of patch occupants	−0.05	−0.15	0.06		0.21	0.01	0.42	
No. of patch occupants^2^	0.01	−0.09	0.12		−0.03	−0.22	0.16	
Mean patch quality	−0.02	−0.11	0.07		0.56	0.25	0.87	
Patch density	0.03	−0.03	0.09		−0.01	−0.07	0.06	

Reference category = ^1^female, ^2^*Acacia erioloba*, ^3^high; Bold variables are those with an importance ≥0.80 and confidence intervals that do not cross zero.

**Table 5:** All-subset averaged models describing the predictors of individuals' patch residency time during foraging in natural and experimental foraging conditions. Importance is measured by Akaike's variable weight

Fixed effects	Natural				Experiment			
	Coefficient	Upper C.I.	Lower C.I.	Importance	Coefficient	Upper C.I.	Lower C.I.	Importance
(Intercept)	4.85	4.29	5.42		4.29	3.80	4.77	
Rank	−0.05	−0.17	0.07	0.93	**0.30**	**0.13**	**0.48**	**1.00**
Social bond strength	0.04	−0.13	0.22	0.96	0.01	−0.18	0.19	0.78
Social bond balance	−**0.23**	−**0.35**	−**0.11**	**1.00**	−0.11	−0.34	0.13	0.73
Relatedness	0.00	−0.14	0.13	0.98	0.01	−0.14	0.16	0.73
Rank × Social bond strength	−0.13	−0.25	−0.02	0.73	−0.12	−0.24	0.01	0.54
Rank × Social bond balance	−0.06	−0.16	0.03	0.44	−0.05	−0.20	0.09	0.26
Rank × Relatedness	−0.04	−0.16	0.08	0.32	−0.11	−0.24	0.01	0.46
Social Bond Strength × Relatedness	0.12	0.01	0.22	0.77	0.02	−0.10	0.14	0.17
Social Bond Balance × Relatedness	**0.12**	**0.02**	**0.22**	**0.83**	0.06	−0.08	0.20	0.22
Social bond strength × Social bond balance	0.07	−0.04	0.18	0.45	0.03	−0.16	0.22	0.19
Initial intake rate	**0.26**	**0.18**	**0.33**	**1.00**	−0.04	−0.17	0.09	0.95
Initial intake rate × Rank	0.04	−0.04	0.12	0.35	**0.19**	**0.05**	**0.33**	**0.89**
Initial intake rate × Social bond balance	**0.11**	**0.04**	**0.19**	**0.98**	0.03	−0.16	0.22	0.23
Initial intake rate × Social bond strength	–	–	–		0.05	−0.06	0.17	0.27
Age	0.02	0.00	0.05		0.04	−0.01	0.08	
Sex (male)^1^	−0.29	−0.69	0.11		0.18	−0.28	0.64	
Satiation	–	–	–		0.05	−0.10	0.20	
Patch quality	0.30	0.17	0.43		0.86	0.52	1.20	
Patch depletion	–	–	–		−0.03	−0.18	0.12	
Patch species^2^	–	–	–		–	–	–	
*Faidherbia albida*	−1.17	−1.82	−0.52		–	–	–	
Herb/shrub	−1.34	−2.22	−0.45		–	–	–	
*Prosopis glandulosa*	−0.88	−1.98	0.23		–	–	–	
*Salvadora persica*	−0.89	−1.79	0.02		–	–	–	
*Tapinanthus oleifolius*	−0.35	−1.25	0.55		–	–	–	
*Acacia tortilis*	−1.53	−2.82	−0.23		–	–	–	
Handling time (low)^3^	0.41	−0.33	1.15		–	–	–	
No. of patch occupants	0.54	0.29	0.79		0.34	0.00	0.69	
No. of patch occupants^2^	−0.38	−0.68	−0.08		0.03	−0.30	0.37	
Time in previous patch	0.04	−0.03	0.11		0.02	−0.08	0.13	
Mean patch quality	−0.15	−0.29	−0.02		−0.80	−1.16	−0.44	
Patch density	−0.22	−0.31	−0.13		0.04	−0.09	0.17	

Reference category = ^1^female, ^2^*Acacia erioloba*, ^3^high; Bold variables are those with an importance ≥0.80 and confidence intervals that do not cross zero.

**Figure 5:**
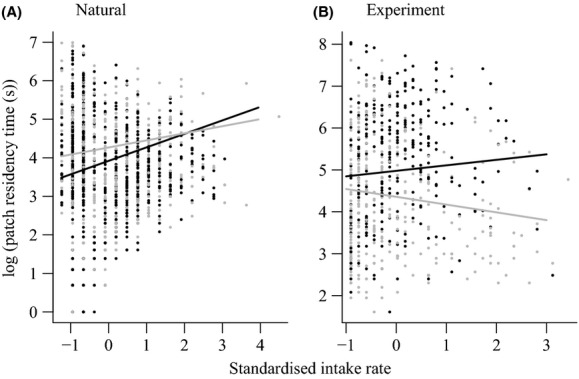
The relationship between initial intake rate and patch residency time in (A) natural and (B) experimental foraging conditions. In both plots lines are the relationships estimated by the models (see Table5) and each point represents a patch visit. The data are split into individuals whose had higher (black) or lower (gray) social bond balance (A) and rank (B) than the median. Patch residency time are plotted on a natural log scale for ease of comparison, and intake rates were standardized to have a mean of zero and standard deviation of one before model fitting.

The baboons experienced median patch residency times of 46 s (interquartile range = 23–97, *n* = 1768) in natural conditions and 112 s (35–377, *n* = 627) in experimental conditions. The initial intake rate a baboon experienced upon entering a patch, its social bond balance, and the interaction between the two were the most important predictors of patch residency time in the natural conditions (Table5). Patch residency times were longer in patches where baboons had experienced higher initial intake rates, and this correlation was more positive (indicating more efficient foraging) in individuals with high social bond balance (Fig.5A). In the experiment, rank and the interaction between rank and initial intake rate were important predictors of patch residency time (Table5). Higher-ranked individuals experienced longer patch residency times and a more positive correlation between their initial intake rate and patch residency time, indicative of more efficient foraging (Fig.5B).

## Discussion

Our study makes two particular contributions. First, it suggests that individuals are able to mitigate foraging costs of low rank, but that the effectiveness of this mitigation may be limited to certain environments. In both conditions, low-ranked individuals received more agonism, but this only translated into reduced foraging performances for low-ranked individuals in the high-competition experimental conditions. Our results suggest one possible reason for this pattern may be the strategic investment of social effort to negotiate foraging tolerance, whose rank-offsetting effect was overwhelmed in the high-competition experimental environment. High social bond balance was associated with a reduction in the agonism received amongst low-ranked individuals in both conditions. However, social bonds' ability to negotiate foraging tolerance is known to be limited by the time available for socializing, and it appears these limits may have been exceeded in the experiment. Second, our study suggests that social bond balance influences foraging behavior in a wide range of environmental conditions, whereas the influence of social bond strength may be reserved for more extreme conditions. Social bond balance influenced the foraging agonism that the baboons received in both conditions, whereas social bond strength only affected foraging agonism in the high-competition experimental environment.

### The effect of rank in the natural and experimental environments

Lower-ranked individuals received relatively more agonism than high-ranked individuals under natural foraging conditions. However, contrary to expectations (Barta and Giraldeau 1998; Smith et al. 2001; Stillman et al. 2002), this did not mean that high-ranked individuals enjoyed markedly greater foraging performances. This suggests that low-ranked foragers were able to mitigate their “poor” social position and maintain similar foraging performances to their higher-ranked counterparts. Mitigation tactics by subordinate foragers have been shown elsewhere (Bugnyar and Kotrschal 2004; Held et al. 2010). Our results suggest that one such mitigation tactic employed by low-ranked baboons may have been the strategic investment of social effort to negotiate foraging tolerance (Barrett et al. 1999; Fruteau et al. 2009). Low-ranking individuals with high social bond balance, that is, individuals who had invested a relatively high amount of time in grooming others, were less likely to receive agonism during foraging.

Previous studies have also shown that increased investment in time grooming others allows individuals to negotiate access better foraging opportunities (*sensu* biological market theory: Noë and Hammerstein 1995; e.g., Barrett et al. 1999; Fruteau et al. 2009). However, they have also shown that social bonds, and particularly their ability to negotiate foraging tolerance, are expected to be limited by the amount of time, and effort individuals can invest in socializing (Barrett et al. 1999; Lehmann et al. 2007; Dunbar et al. 2009; Fruteau et al. 2009). Our experiment, where agonism was more frequent, appears to have exposed the limits of individuals' ability to negotiate foraging tolerance through the using imbalances in their social bonds. This suggestion is supported by two pieces of evidence. First, although the agonism received by low-ranked individuals was moderated by social bond balance to a greater extent in the high-competition environment than under natural conditions, high-ranked individuals still achieved greater foraging performances. Second, social bond balance influenced foraging efficiency in the natural but not experimental conditions. Foraging efficiency was expected to be greater in individuals who were less likely to have to leave a patch before the optimum time (and so was measured as the correlation between initial intake rate and patch residency time, Nonacs 2001). Individuals with high social bond balance experienced greater foraging efficiencies in natural conditions, suggesting they were able to negotiate greater foraging tolerance from others. However, this was not the case in the in the experiment, where high-ranked individuals experienced greater foraging efficiencies instead.

We did not, however, find an interactive effect between rank and social bond balance on any measure of individuals' foraging performance. This suggests that mitigation tactics, other than the strategic investment of social effort to negotiate foraging tolerance, were being used which allowed low-ranked individuals with low social bond balance to maintain similar foraging performances to other group members. A candidate tactic is the use of more peripheral and unoccupied patches in the natural conditions. Such a pattern has been observed in many social foragers (Hall and Fedigan 1997; Melletti et al. 2010; Hirsch 2011), including our baboons (King et al. 2009), and would allow these individuals to reduce the foraging competition they experienced but also comes with costs such as increased predation risk and reduced access to social information (Hirsch 2007; Morrell and Romey 2008; Rieucau and Giraldeau 2011). Resources in the experimental environment were spatially clumped in only five patches, and so there was no opportunity for individuals to offset the foraging costs of low rank through the use of more peripheral or unoccupied patches. Therefore, similarly to the strategic investment of social effort by low-ranked individuals, it would be expected that this mitigation tactic would also have been overwhelmed in the experiment.

An alternative explanation to the use of mitigation tactics to offset the foraging costs of low rank may be that in the natural environment foraging costs were low, meaning there were few or no disadvantages of being low-ranked for individuals to offset. However, we consider this scenario unlikely as our results show there were strong rank effects on the agonism individuals experienced during foraging, the levels of agonism we recorded are comparable to other baboon systems where foraging costs of low rank have been demonstrated (e.g., Barrett et al. 2002), and these costs have also been demonstrated previously in our system (King et al. 2009; Marshall et al. 2012b).

Increased spatial clumping of resources (in this case, from natural foraging to experimental foraging conditions) can lead to increases in foraging competition, of which low-ranked foragers disproportionately bear the costs (birds: Stillman et al. 1996; Vahl et al. 2005; primates: Barrett et al. 2002; King et al. 2009). However, it is worth noting that these foraging benefits of high rank may be associated with other costs. For example, in our study high-ranked individuals may have experienced a greater risk of injury and higher energetic costs as a result of the receiving proportionally less (and so initiating more) agonism in both conditions, and being involved in higher overall rates of agonism in the natural conditions. Nonetheless, rank has also been shown to have a more pronounced positive effect on reproductive success during periods of low resource availability, both in baboons (Altmann and Alberts 2003) and other species (e.g., Nichols et al. 2012; reviewed by Clutton-Brock and Huchard 2013), and good quality social bonds can offset the negative effects of low rank on an individual's longevity (Silk et al. 2010b). This growing body of evidence suggests that tactics through which individuals can offset the negative fitness effects of low rank, such as the strategic investment of social effort or the inferred use of unoccupied patches, do exist and are adaptive, but that their offsetting ability is limited to certain environmental conditions.

Such environment-dependent tactics may also help to explain why seasonal variation in resource distribution and foraging competition can lead to transient advantages for high-ranked foragers (Barrett et al. 2002; Stillman et al. 2002). Indeed, where natural environmental variation is lower, tactics that offset the foraging costs of low rank may be difficult to detect as they are rarely overwhelmed. However, our findings indicate that where food distributions remain concentrated over longer periods, low-ranked individuals may have to persistently bear the costs of increased competition with implications for their fitness and, ultimately, the profitability of remaining a member of the social group. Where such resource distributions occur in natural environments, these costs may lead to low-ranked individuals needing to spend more time foraging, restricting the time available for other activities such as socializing below critical minimum limits (Dunbar 1992; Dunbar et al. 2009). Changes in individual time budgets are likely to have group-level consequences (Marshall et al. 2012a) and are thought to contribute to the existence of a maximum tolerable group size an environment can support or, where this ecological maximum is less than the minimum size needed for a group to function, the absence of a species from that environment (Dunbar 1992; Courchamp et al. 1999; Dunbar et al. 2009). Consequently, where anthropogenic impacts such as climate change-induced environmental extremes or urbanization cause a similar consistent concentration of resources, the persistently increased foraging costs on low-ranked individuals that this produces are likely to cause either a reduction in group size or a local extinction of the species from that area.

### The comparative effect of social bond balance and strength in the natural and experimental conditions

Our study found differences in the comparative effect of social bond balance and social bond strength on foraging agonism between the natural and experimental environments. Whilst social bond balance was associated with the foraging agonism in both environments (as discussed above), social bond strength only influenced foraging agonism in the higher-competition experiment. Here, strong social bonds were associated with (1) reduced overall rates of agonism and (2) in low-ranked individuals, the receipt of a greater proportion of these agonistic interactions. This suggests (1) that the main benefit of strong social bonds in the high-competition experimental environment was in lowering the overall level of agonism an individual was involved in. It also perhaps suggests (2) that low-ranked individuals with strong social bonds may have benefited by disproportionately reducing the amount of agonism they initiated, so leading to an increased proportion of agonism received, albeit at a lower rate overall.

The adaptive role of social bonds has been the subject of considerable research effort (Silk 2007; Henzi and Forshaw 2013) and has resulted in two alternative perspectives. Some studies suggest that short-term imbalances in social bonds (in this study “social bond balance”) are traded for commodities such as foraging tolerance in a biological market (Barrett et al. 1999; Fruteau et al. 2009; Tiddi et al. 2011). Other studies, however, suggest that individuals preferentially exchange tolerance with group members with whom they hold long-term social bonds (in this study “social bond strength”) (Crockford et al. 2008; Frank and Silk 2009; Silk et al. 2010a). Our findings may hint at a reconciliation between these two perspectives, supporting a previous study suggesting they need not be mutually exclusive (Silk et al. 2010a). In most conditions, such as our natural environment, short-term imbalances in social bonds may provide enough negotiating power to offset any foraging costs of low rank. This could explain why, in the short term (within years), an individuals' relative contribution to its social relationships often varies considerably, and the identity of some social partners can be quite changeable between years (Gomes et al. 2009; Henzi et al. 2009; Silk et al. 2012). The main function of these relationships may be to negotiate access to resources, such as food in this study. Indeed, a recent study at our site has found that individuals' grooming contributions can vary strategically across a day (Sick et al. 2014). In contrast, the role of maintaining long-term (across years) and balanced relationships with other individuals may be to provide social support over longer time scales (Gomes et al. 2009; Silk et al. 2010a, 2012), with these relationships only exploited to provide short-term tolerance when the negotiating power of short-term relationships is exhausted, such as during periods of high foraging competition as in the experimental conditions.

Our finding that individuals with strong social bonds were associated with lower initial intake rates under natural conditions is surprising and contradicts previous studies which have generally found a positive effect of social bonds on foraging behavior (e.g., Fruteau et al. 2009; King et al. 2009). This effect may be because we were only able to measure intake rates at the start of a patch visit when foragers would be collecting social and personal information about the patch's quality (Dall et al. 2005). The access and use of social information has been linked to individuals' social bonds (Voelkl and Noë 2008; Aplin et al. 2012). Individuals with strong social bonds may have been monitoring other patch occupants' behavior and so reducing their initial intake rate. Individuals with weaker social bonds may have been relying more on personal information about the patch quality, which they would have collecting through directly sampling it. It may be that social bond balance did not have a similar effect on initial intake rates in natural conditions as its main role is in negotiating foraging tolerance rather than gaining access to social information (and so had a weakly positive effect in initial intake rates).

## Conclusion

This study highlights that the effect of social traits such as rank and social bonds on individuals' foraging behavior can depend on environmental conditions. It suggests that mitigation tactics may allow individuals to offset the foraging costs of low rank and that the strategic investment of social effort to negotiate foraging tolerance and the possible use of peripheral food patches may be ways individuals achieve this. However, it appears that where resources are concentrated creating particularly intense competition, perhaps due to high levels of seasonality, extreme climatic events or anthropogenic environmental change, the limits of these mitigation tactics can be exceeded, resulting in reduced foraging performances for low-ranked individuals.
